# The role of complement in normal pregnancy and preeclampsia

**DOI:** 10.3389/fimmu.2025.1643896

**Published:** 2025-07-24

**Authors:** Richard M. Burwick, Anuja Java, Jean F. Regal

**Affiliations:** ^1^ Maternal Fetal Medicine, San Gabriel Valley Perinatal Medical Group, Pomona Valley Hospital Medical Center, Pomona, CA, United States; ^2^ Division of Nephrology, Department of Medicine, Washington University School of Medicine, St. Louis, MO, United States; ^3^ Department of Biomedical Sciences, University of Minnesota Medical School, Duluth, MN, United States

**Keywords:** innate immunity, complement, pregnancy, preeclampsia, pregnancy loss, fetal development

## Abstract

Preeclampsia affects 3-4% of pregnancies with adverse effects for both mother and child. Minimal therapeutic options are available, and biomarkers are urgently needed to identify those at greatest risk early in the pregnancy. Both the innate and adaptive immune systems are well regulated during normal pregnancy including the complement system. A functioning complement system with some degree of complement activation participates in proper placental development, ensuring a healthy pregnancy and assisting with host defense. However, aberrant complement activation can lead to adverse pregnancy outcomes such as preeclampsia. An overview of the complement system will be presented, along with review of the pre-clinical literature in animal models providing evidence for complement involvement in maintaining a normal pregnancy and contributing to symptoms of preeclampsia. In addition, clinical studies with evaluation of complement biomarkers in plasma and urine implicate complement dysregulation in the pathophysiology of subtypes of preeclampsia including HELLP (hemolysis, elevated liver enzymes and low platelet count) syndrome. Recent studies on the genetics of complement dysregulation in preeclampsia will be reviewed, along with updates on use of recently developed complement therapeutics. The potential utility of evaluating complement activation or manipulating complement during pregnancy will be discussed in view of the successful use of complement therapeutics in pregnancy in other immune diseases.

## 1 Introduction

From an evolutionary perspective, the complement system is remarkably well conserved, underscoring its vital role in survival. This importance is further highlighted by ‘natural experiments’ of complement deficiencies that reveal the essential contributions of the system’s more than 50 proteins. In the absence of an intact complement system, or when the system is dysregulated, there is an increase in susceptibility to infections and immune complex diseases. Even more nuanced are genetic variants in complement components that can impair function and increase risk of infections, kidney disease, autoimmune disorders and adverse pregnancy outcomes.

Pregnancy requires very careful regulation of both the innate and adaptive immune system. Extensive research has shown that the complement system, an essential bridge between innate and adaptive immunity, plays a critical role in maintaining healthy pregnancy. While a fully functional complement system is necessary for normal gestation, its excessive activation is detrimental and has been strongly associated with preeclampsia. This illustrates the “double -edged sword” nature of the complement system. This review will focus on preeclampsia and hypertensive disorders of pregnancy, exploring the role of complement in their pathophysiology. Understanding the system’s contribution to normal pregnancy provides essential context for these disease processes.

The goal of this review is to provide a comprehensive overview of the complement system, both canonical and non-canonical pathways, with particular emphasis on its role at the maternal-fetal interface. We will review evidence from human studies and animal models that demonstrates how complement activity influences various stages of pregnancy and contributes to preeclampsia. Additionally, we will discuss how experimental manipulation of complement pathways in disease states has informed the safety and therapeutic potential of targeting complement during pregnancy. Finally, we will explore recent genetic advances that shed light on how variations in complement regulation can increase the risk of adverse pregnancy outcomes.

## 2 Complement system

The complement system was first described by Bordet as a heat-labile component of blood that enhanced the bactericidal activity of antibodies, a discovery that earned him the Nobel Prize in 1919. Decades of subsequent research and advances in protein biochemistry have led to our understanding of the biochemistry of the complement activation pathways that we teach to aspiring physicians; the classical, lectin and alternative pathways. However, continuing studies have significantly expanded our understanding of the complement system’s role. Beyond its established functions in host defense, complement also plays a critical role in maintaining homeostasis through mechanisms that extend outside the traditional activation cascades. These so-called non-canonical activities of complement, occurring both extracellularly and within cells, have become an area of growing interest and importance in immunology.

### 2.1 Extracellular (canonical and non-canonical)

The canonical complement system in the blood has been extensively reviewed and is a key chapter in all immunology textbooks. The complement system is important in protecting from infection and in clearance of immune complexes as evidenced by evaluation of individuals with deficiencies in various complement components. The reader is pointed to some very comprehensive recent reviews of complement for a more detailed look at the system (1–3).

**Figure 1:**
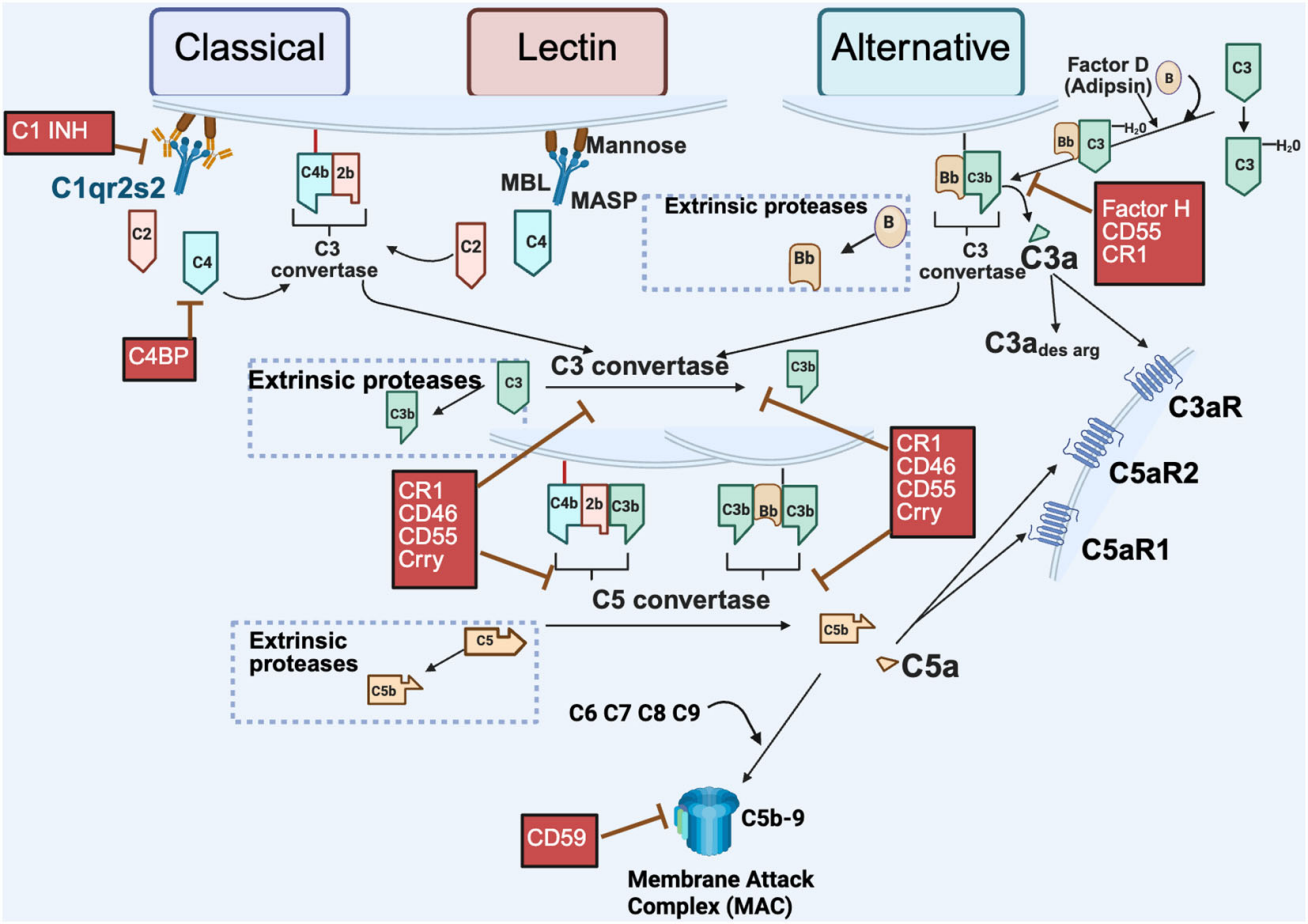
Extracellular pathways of complement activation. Complement activation via the *canonical* pathways (classical, lectin and alternative) are depicted with distinct initiation steps. The classical pathway is initiated by antigen interaction with specific antibody to activate the C1qr2s2 molecule (C1 esterase) cleaving C4 and C2 resulting in a bimolecular enzyme complex C4b2b, the classical pathway C3 convertase. The lectin pathway is initiated by carbohydrates on invader surfaces binding to MBL (Mannose binding lectin) and engaging of Mannose associated serine proteases (MASP 1 & 2) to generate the identical bimolecular enzyme complex C4b2b. The alternative pathway can be activated by foreign surfaces in conjunction with the constant low level C3 tickover [C3 to C3(H_2_0)]. In addition, cleavage of C3, C5 and Factor B can occur in the extracellular environment by extrinsic proteases such as trypsin, thrombin, and kallikrein – *non-canonical* activation (dotted boxes). Fragments generated from the action of extrinsic proteases can result in effector functions or continued pathway activation from that point. Depicted in red are select regulators of the complement pathway; fluid phase (Factor H, C1 INH, and C4BP) or membrane bound (CR1, CD46, CD55, CSMD and CD59, rodent specific Crry) that limit complement activation at the crucial C3 or C5 convertases or other points in the pathway. Created in BioRender. (2025) https://BioRender.com/ad285s3.

As illustrated in **Figure 1**, complement activation occurs through three major pathways: the classical, lectin and alternative. Each pathway is initiated by a distinct mechanism, but they share the common goals of target recognition and opsonization, promoting the inflammatory response as well as lysis of abnormal cells or pathogens. The classical pathway is typically initiated by antibodies bound to antigens, which recruit the C1 complex (C1q, C1r, C1s—collectively called C1 esterase). This leads to cleavage of C4 and C2, generating the classical pathway C3 convertase, C4b2b, with C4b covalently attached to activating surfaces. *Note*: There is ongoing debate among complement experts regarding nomenclature. Older literature refers to the larger fragment of C2 as C2a, while newer conventions prefer C2b to maintain consistency across the cascade (4). The lectin pathway is triggered by recognition of specific carbohydrate patterns on pathogens. Mannose-binding lectin (MBL), collectins and ficolins bind these carbohydrates and activate MASPs (MBL-associated serine proteases), leading to the same C3 convertase (C4b2b) as in the classical pathway. The alternative pathway, distinct from the classical pathway, has the capacity to deposit on a pathogen without need for prior contact/exposure. It relies on the slow, continuous hydrolysis of C3 in plasma, a process known as “tickover”, to form C3(H_2_O). Upon encountering an activating surface, C3b is generated and binds Factor B, which is then cleaved by Factor D (also known as adipsin), resulting in the formation of the alternative pathway C3 convertase, C3bBb. C3, present in high serum concentrations (1–2 mg/ml), is cleaved by either C4b2b or C3bBb. The resulting C3b fragment binds covalently to pathogens or proteins, promoting opsonization and further amplification of the cascade. Addition of another C3b to each C3 convertase forms the C5 convertases (C4b2b3b and C3bBbC3b), which cleave C5 into C5a and C5b, the final proteolytic step in complement activation. C5a and C3a, small molecular weight anaphylatoxins, bind to their respective G-protein-coupled receptors (C5aR1, C5aR2, and C3aR1), eliciting pro-inflammatory responses. C5b, in turn, initiates assembly of the membrane attack complex (MAC, C5b-9), leading to target cell lysis.

Complement activation is tightly controlled by both membrane-bound and fluid phase regulator proteins to prevent host tissue damage. In the classical pathway, C1 inhibitor (C1INH) and C4 Binding Protein (C4BP) are soluble factors that block early steps by targeting C1 and C4, respectively. Factor H (FH), a key fluid-phase regulator, primarily controls the alternative pathway C3 convertase by promoting the cleavage of C3b (known as cofactor activity) and accelerating convertase decay (known as decay accelerating activity). CD55 (Decay-accelerating factor, DAF) promotes dissociation of the convertases, while CD46 (Membrane cofactor protein, mCP) serves as a cofactor for the Factor I (FI)-mediated proteolytic inactivation of C3b and C4b deposited on cells. Both CD55 and CD46 are widely expressed in human tissues and regulate all three pathways. CR1 (CD35, C3b/C4b receptor) present on human erythrocytes has cofactor activity and decay accelerating activity for both the classical and alternative pathways. CD59 (Protectin), another membrane-bound regulator, prevents MAC formation by inhibiting C9 polymerization. Other regulators like CSMD1 and CSMD3, though less well characterized, may have cofactor activity or effects on MAC, and are also implicated in neurological processes (5–7).

Species-specific complement differences complicate the study of the role of complement in normal pregnancy and preeclampsia. For example, Crry (CR1-related gene/protein Y) in rodents (but not humans) performs functions analogous to both CD46 and CD55 in humans. In mice and rats, CD46 is only expressed in the testes suggesting a specialized role in reproduction in comparison to a more global role and distribution for CD46 on human cells. Thus, use of animal models to investigate a role for CD46 in pregnancy is not readily translatable to human pregnancy. Activators of the alternative pathway also differ between species. For example, rabbit erythrocytes activate the alternative pathway in human serum, but not rabbit (8) and thus are used to assay alternative pathway activation in human serum. Zymosan however activates the alternative pathway in multiple species. Human CD59 can protect against cell lysis by human complement, but not by rat and guinea pig complement (9). Eculizumab is an anti-C5 antibody therapeutically effective in the human but does not react with mouse C5. The mouse monoclonal antibody BB5.1 has been used for proof-of-concept studies in mice to accumulate evidence for the importance of C5. Also, novel anti-C5 monoclonal antibodies capable of inhibiting complement in multiple species have been developed to assist in preclinical studies to evaluate complement involvement (10) and are useful tools for proof-of-concept studies in rat, but with low reactivity in mouse. ELISA assays developed for measurement of human complement components and activation products are not guaranteed to be accurate for measurement of mouse or rat complement, so quantitation of components in pre-clinical studies must be carefully planned and validated, along with effectiveness of inhibitors. These differences pose challenges in translating animal model findings to human pregnancy and diseases such as preeclampsia.

Complement receptors mediate the downstream effects of activation products. CR1 (CD35) binds C3b and C4b, along with their breakdown products, iC3b and iC4b, and plays a major role in clearing immune complexes, especially in primates (11). C3a and C5a, potent inflammatory mediators, signal through G protein coupled receptors C3aR and C5aR1/2. Notably, C3a can be converted to C3a_des arg_ (also called ASP, acylation-stimulating protein) and C5a to C5a_des arg_ via carboxypeptidase cleavage. While C3a binds C3aR and C5a can interact with C5aR1 and C5aR2, both C3a and ASP have been reported to interact with C5aR2 on adipocytes to promote triglyceride synthesis, though this interaction is controversial (12, 13) due to the conflicting findings regarding the exact nature and significance of this interaction. While the initial studies identified ASP as a potential ligand for C5aR2 that stimulates triglyceride synthesis in adipocytes, other studies provided contradicting evidence. Therefore, further research is needed to fully understand the intricate relationship between C3a/ASP, C5aR2, and their roles in adipocyte function and metabolic regulation.

Beyond traditional pathways, complement components can also be activated by extrinsic proteases, a process independent of convertases. Proteases from the coagulation, fibrinolytic, and kallikrein-kinin cascades (e.g., plasmin, thrombin, kallikrein) can directly cleave C3, C5, and Factor B, generating active fragments such as C3b, C3a, C5b, C5a, and Bb. These fragments can then initiate effector responses themselves or go on to assemble convertases. This highlights the complement system’s versatility: both as a tightly coordinated cascade and as a modular set of components that contribute to immune surveillance, inflammation, and tissue homeostasis.

### 2.2 Intracellular

**Figure 2:**
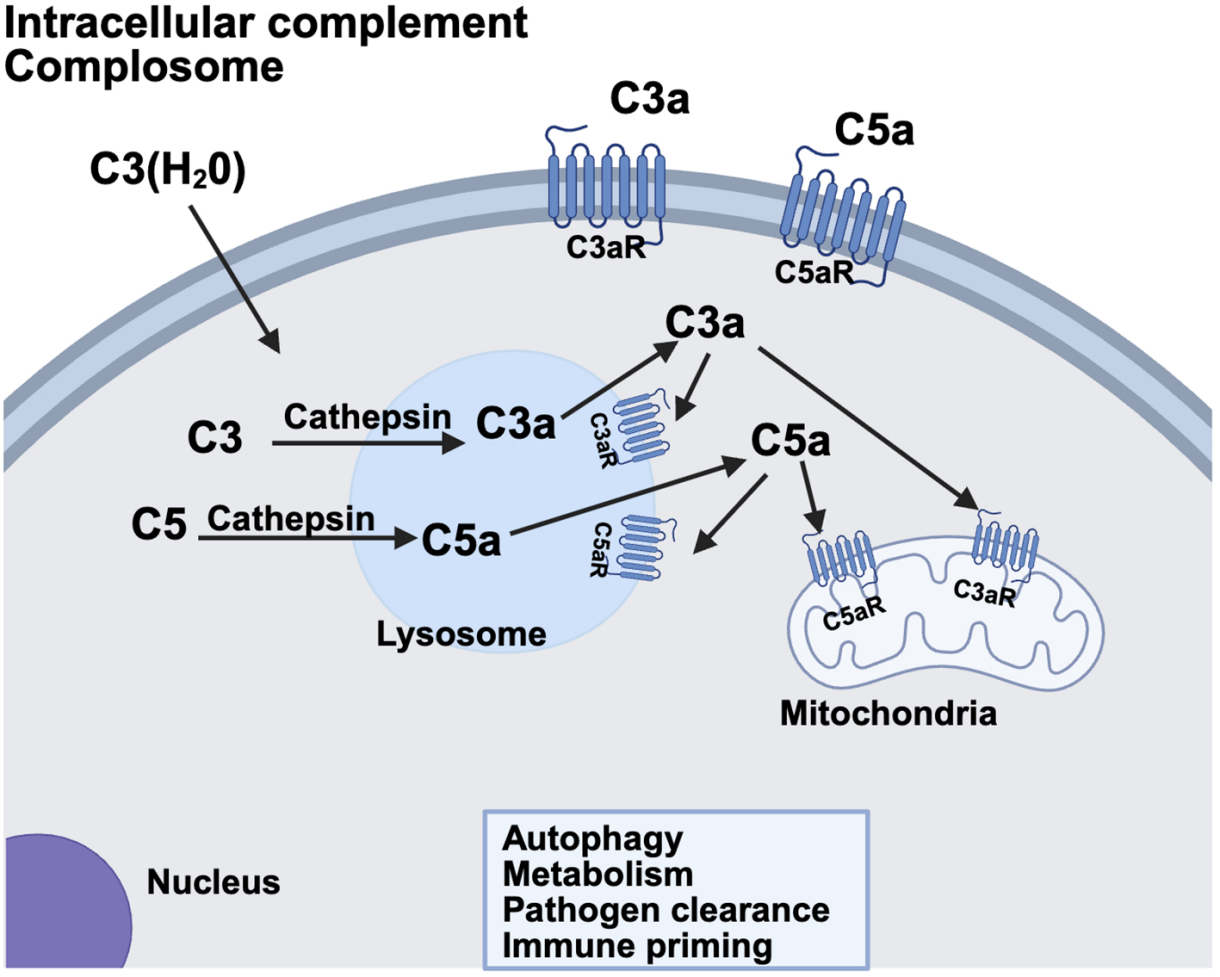
The non-canonical intracellular complement system. Complement components C3 and C5 are synthesized within the cell or shuttled into the cell in some cases. Lysosomal proteases such as cathepsin can then cleave the molecule yielding C3a/C3b or C5a/C5b, respectively, within the cell. C3a and C5a receptors have been identified within the cell on lysosomes and mitochondria. Alternatively, there is some evidence that C3a/C5a generated intracellularly can then act in an autocrine fashion on plasma membrane C3a/C5a receptors. Intracellular complement has been identified and characterized in T cells, Beta cells of the pancreas and the CNS. Ongoing research is identifying complement components operative in autophagy, metabolism, pathogen clearance and immune priming of cells in many different systems and cell types. Created in BioRender. (2025) https://BioRender.com/u1u2zs3.

The intracellular complement system, also referred to as the ‘complosome,’ has garnered growing attention for its roles in maintaining cellular homeostasis and modulating local innate immune responses (**Figure 2**). Although it has been known for decades that complement proteins are synthesized by lymphocytes and other immune cells outside the liver (14), recent studies have revealed previously unrecognized functionality of this system within cells. This was first demonstrated in T cells (15), and has since been extended to the central nervous system (16–18) and the pancreatic islets (19–21) where intracellular complement activation products contribute to both intracellular signaling and autocrine effects in the local microenvironment. Emerging evidence suggests that the tickover product C3(H_2_0) can be shuttled from the extracellular space into the cytosol (22), where it is cleaved in lysosomes by Cathepsin L to generate C3a and C3b. Intracellular C3a interacts with the G protein coupled receptor C3aR either within the cell or at the plasma membrane, to evoke effector responses. Meanwhile, C3b covalently binds to membrane surfaces, tagging them for immune recognition. There is also evidence that C5 is cleaved in the lysosome to form C5a and C5b. C5a then engages C5aR1 on lysosomes and mitochondria, influencing key cellular processes such as metabolism, pathogen clearance and regulation of autophagy. The specific intracellular complement components vary across different cell types, and this area remains an active and rapidly expanding field of study. At the fetal-maternal interface, for example, trophoblasts are known to synthesize complement components such as C4, C3 and terminal components (23), while endothelial cells in the spiral artery produce C1q (24). The precise roles of these locally synthesized or intracellularly active complement components in supporting normal pregnancy outcomes are currently under intense investigation.

## 3 Preeclampsia

A key concept in understanding preeclampsia is that it is not a single disease but a syndrome or group of symptoms with diverse underlying mechanisms. Preeclampsia has been strongly linked to the placenta because the only definitive ‘cure’ for preeclampsia is delivery of the placenta. It is increasingly recognized that the pathophysiology of preeclampsia is heterogeneous and may differ depending on the subtype, symptom in question and the severity and organ involvement. Preeclampsia falls within the broader spectrum of hypertensive diseases of pregnancy, which includes gestational hypertension, preeclampsia, and superimposed preeclampsia. These conditions can potentially lead to more severe manifestations such as eclampsia or HELLP syndrome (hemolysis, elevated liver enzymes and low platelet count). As shown in **Table 1**, clinical features often overlap across subtypes and may differ subtly. Preeclampsia is considered severe when blood pressure is greater than or equal to 160/110 mmHg, or when signs of end-organ damage are present. HELLP syndrome, a form of thrombotic microangiopathy (TMA), is diagnosed by the presence of microangiopathic hemolysis, elevated liver enzymes and low platelet count (25). Maternal symptoms commonly include right upper quadrant abdominal pain, nausea, vomiting and fatigue (26). HELLP syndrome is the most common TMA in pregnancy, affecting approximately 0.5%-1% of all pregnancies. Over two-thirds of cases occur in the third trimester, though some present in the immediate postpartum period (27). Fetal growth restriction often accompanies hypertensive disorders of pregnancy but is not essential to the definition (28). Nonetheless, it is frequently used as a marker in animal models to simulate preeclampsia, particularly in the context of impaired placental perfusion.

**Table 1:** Subtypes of hypertensive disorders of pregnancy and distinguishing characteristics.

Subtype	Hypertension	Distinguishing characteristics
Gestational Hypertension	+	No proteinuria or systemic symptoms ^b^
Early Onset Preeclampsia (EOP)	+	Hypertension appears at 20–34 wk gestation with proteinuria and/or systemic symptoms
Late Onset Preeclampsia (LOP)	+	Hypertension appears at >34 wk gestation with proteinuria and/or systemic symptoms
Superimposed Preeclampsia	+	EOP or LOP with preexisting hypertension prior to pregnancy or before 20 wk gestation
HELLP ^a^ syndrome	+/-	Hemolysis, elevated liver enzymes, low platelets, thrombotic microangiopathy (TMA) after 20 wk gestation
Eclampsia	+/-	Seizures, occurring in the setting of preeclampsia or HELLP syndrome, and not explained by underlying seizure disorder

a Hemolysis, elevated liver enzymes, low platelet counts.

b Systemic symptoms can include proteinuria, severe headache, blurred vision, pain in upper right abdomen, pulmonary edema, oliguria, liver enzyme elevation (>2x normal), serum creatinine >1.1mg/dl, or thrombocytopenia (platelet count <100 k/ul).

Is complement critical for the pathophysiology in all clinical scenarios? Certainly not. The biological drivers of disease likely vary across different clinical scenarios. More effective therapies will come from tailoring treatment to the specific underlying etiology. A better understanding of the evidence implicating complement in both human studies and animal models will help guide rational, targeted interventions, administered at the right time and to the right patient.

## 4 Complement in pregnancy and preeclampsia

### 4.1 Complement regulation at maternal-fetal interface is essential for favorable pregnancy outcome

A favorable pregnancy centers on the placenta, a unique temporary organ formed in the uterus to connect mother to the developing fetus. Many adverse pregnancy outcomes result from defective placentation. Although placental structure varies across species, the critical sites for complement system regulation may be conserved or differ slightly. In humans, rats and mice, the placenta is hemichorial i.e. the fetal epithelium is in direct contact with the maternal blood (29). Rodent placental layers are termed decidual, junctional and labyrinth whereas layers in the human placenta have different terminology (extravillous, extravillous trophoblast column, villous). Again, recent reviews provide excellent background of the details (30). The other key feature of the placenta is that the decidual layer is maternal and the trophoblasts are fetal (both mom and dad). This fetal-maternal interface represents a potential immunological challenge, making complement regulation crucial to prevent maternal rejection of the allogeneic fetus. In rodents, three layers of trophoblasts, including two syncytiotrophoblasts and one cytotrophoblast, separate maternal blood from fetal capillaries, whereas in humans, only a single syncytiotrophoblast layer is present. Trophoblasts invade the maternal decidua to remodel uterine spiral arteries to a low resistance blood vessel. This invasion is deeper in rats and humans than in mice. Given these differences, complement regulation is particularly important at two sites: the point of trophoblast invasion into the spiral arteries and the syncytiotrophoblast layer where maternal blood directly contacts fetal tissue. Smith Jackson’s review offers a comprehensive look at complement’s role in placental development, emphasizing the importance of balancing complement activation with regulation (31).

Pregnancy represents an allogeneic-type mismatch between the fetus and the maternal immune system (32, 33). Since the fetus encounters maternal immunocompetent cells during trophoblast invasion, healthy placentation depends on a collaboration between efficient trophoblast invasion and the mother’s immune system. During trophoblast invasion, many immune cells infiltrate the decidua, allowing them to reach the endometrium and spiral arteries. In healthy pregnancies, non-recognition of these trophoblasts by maternal immune cells promotes maternal-fetal tolerance. The complement system regulates key processes to establish and maintain maternal-fetal tolerance during a healthy pregnancy (34, 35). Placental damage in the human is prevented by fluid phase and membrane bound regulators of complement activation, such as, DAF (CD55), MCP (CD46), CD59 (36), FH, FI, or complement receptor 1 (CR1) (37). These regulators assist in the clearance of placental fragments that enter the maternal circulation as a result of syncytiotrophoblast turn-over. Furthermore, levels of many complement proteins increase during pregnancy. Improper clearance of such components, driven by an inadequately regulated complement system, may cause deposition of debris in tissues and vascular walls, leading to an overly exuberant inflammatory response, resulting in endothelial injury and placental dysfunction (38). Complement dysregulation or overactivation can further activate programmed death of trophoblasts, known as apoptosis, significantly disrupting their migration and placental vascularization. This in turn can lead to placental ischemia and release of inflammatory mediators by the placenta. Due to its potent and rapid nature, complement activation at the fetal maternal interface requires strict control for a successful pregnancy.

### 4.2 Complement and angiogenic imbalance in preeclampsia

In women who develop preeclampsia, early impairments in placental blood flow and oxygenation, often due to poor spiral artery remodeling (39, 40) lead to placental inflammation and apoptosis. These changes lead to systemic activation of leukocytes and endothelial cells over the course of pregnancy (41, 42). Prior to the onset of clinical disease, there is an increase in the release of soluble fms-like tyrosine kinase-1 (sFLT1) by trophoblasts. sFLT1 acts as an anti-angiogenic factor which binds and sequesters placental growth factor (PlGF) and vascular endothelial growth factor (VEGF) (43–45). Elevated sFLT1 levels, along with decreased PlGF and VEGF, are associated with hypertension and proteinuria, and eventual development of preeclampsia (45, 46).

Angiogenic imbalance and excessive complement activation are both associated with development of preeclampsia and abnormal placentation, but whether angiogenic imbalance results in complement activation or vice versa is unresolved. Banadakoppa et al. demonstrated that increased complement activation in trophoblasts correlates with elevated secretion of sFLT1 protein (47). As complement activation increases, intracellular sFLT1 levels decline due to enhanced secretion of sFLT1 protein from trophoblast cells. Notably, this secretion closely correlated with trophoblast release of C5b-9. Supporting this, Collier et al. found strong spatial and quantitative associations between placental C5b-9 and sFLT1, with sFLT1 localization observed near immunofluorescence signals for C4d and C5b-9 on the syncytiotrophoblast membrane (48). *In vitro* studies with human umbilical vein endothelial cells (HUVECs) (49) demonstrated that addition of PlGF increased the expression and secretion of FH, a key complement regulatory protein. In contrast, sFLT1 decreased FH levels making the HUVECs more susceptible to complement activation. These findings suggest that an imbalance in angiogenic factors may enhance complement activation by downregulating complement regulation via FH.

Studies exploring the relationship between angiogenic imbalance and excessive complement activation have also been conducted in preclinical animal models as understanding of the sequence of events preceding fetal loss, growth restriction and adverse maternal consequences could have important ramifications for therapeutic development. In the rat model of placental ischemia induced hypertension (50), inhibition of complement activation from GD14–19 resulted in attenuation of the maternal hypertension, but had no effect on the reduced VEGF or reduced fetal weight. These data suggest complement activation was not critical for the reduction of VEGF in late pregnancy within this model. The BPH mouse model, which exhibits pre-pregnancy borderline hypertension, obesity, and dyslipidemia, is considered a relevant model of superimposed preeclampsia (51). During pregnancy, BPH dams develop high blood pressure, fetal loss, growth restriction and abnormal placentation, features characteristic of preeclampsia. Work by Sones et al. showed complement activation in this model recruits neutrophils very early in gestation at e8.5 leading to increased tumor necrosis factor (TNF), reduced placental VEGF, abnormal placentation and fetal death by e12.5. Complement inhibition restored VEGF levels, suggesting that complement activation leads to angiogenic imbalance (51) and adverse pregnancy outcomes. However, further analysis suggests a more complex picture. In the BPH model, dysregulated VEGF pathway genes occurred at the start of decidualization (e5.5) and precedes the complement deposition in the placenta seen at the end of decidualization and beginning of placentation (e7.5) suggesting that angiogenic dysregulation may initiate complement activation rather than result from it (52). Similarly, in the abortion prone CBA/J X DBA/2 mouse model characterized by fetal rejection and growth restriction without maternal hypertension (53), blocking of complement activation reduced sFLT1 levels and rescued fetal growth. Taken together, these studies indicate that the relationship between angiogenic imbalance and complement activation may differ depending on the stage of pregnancy and the model. The two processes may operate in parallel or redundantly rather than in a strictly linear fashion. Overall, evidence suggests that in early gestation, angiogenic imbalance likely precedes and contributes to complement activation, impairing placental development. In contrast, during later stages, complement activation may be more directly involved in adverse maternal outcomes such as hypertension, independent of angiogenic imbalance. Continued studies in animal models are needed to further clarify these complex interactions.

## 5 Complement components in pathophysiology of preeclampsia

### 5.1 C1q and MBL

Studies by Agostinis in human trophoblasts have demonstrated the importance of C1q in the trophoblast invasion of the spiral arteries and their remodeling for proper placental development. This non-canonical effect of C1q does not require pathway activation. C1q is synthesized by endothelial cells in the decidua as well as by endovascular trophoblasts and macrophages. It facilitates connections between endothelial cells and endovascular trophoblasts, contributing to effective spiral artery remodeling during placentation (24, 54).

Mice deficient in complement components or regulators have revealed extensive information about the role of complement in pregnancy and preeclampsia (55). In the C1q-deficient mouse, trophoblast invasion into decidua is impaired, resulting in hallmark features of preeclampsia, including hypertension and reduced fetal weight (56–58). These effects occur despite the absence of overt pathway activation, further supporting a structural or signaling role for C1q. Mating experiments revealed that only when wild-type (WT) females were mated with C1q-deficient males did preeclampsia-like outcomes manifest, including fetal demise and maternal hypertension. Since the placenta is influenced more by paternal genes, WT females carrying heterozygous (C1q-deficient) placentas developed all major symptoms of preeclampsia. In contrast, C1q-deficient females mated with WT males exhibited normal pregnancies, indicating that the presence of C1q on fetal-derived placental trophoblasts, rather than maternal C1q, is critical for a healthy pregnancy (56). Another key finding in these studies is that the impaired placental development in the absence of C1q leads to reduced placental blood flow and placental ischemia along with secondary mechanisms of tissue injury and inflammation with endothelial damage. The WT females mated with C1q deficient males had greater generation of C5a and other inflammatory mediators later in pregnancy compared to a normal pregnancy suggesting exaggerated canonical complement activation also occurred following the impaired placental development due to C1q deficiency (58). In addition, the WT females mated with C1q deficient males maintained high blood pressure 2 months postpartum when fetal-derived placental trophoblasts lacking C1q were clearly not present. Sutton et al. expanded these findings by investigating the vascular dysfunction postpartum of wild-type (WT) females mated with C1q-deficient males. In this study, vascular dysfunction persisted up to 6 months postpartum, emphasizing a very lasting impact of a compromised placenta on maternal cardiovascular health following pregnancy (59).

Immunohistochemical analyses of placentae from preeclamptic women has also shown that deposition of complement C1q differed significantly between patients with preeclampsia and controls and further demonstrated that early-onset preeclampsia patients had more C1q deposition than late-onset preeclampsia patients (60). This C1q deposition in the syncytiotrophoblast region was thought to reflect complement activation from placental dysfunction rather than the non-canonical function of C1q bridging trophoblasts and endothelial cells in spiral artery remodeling. Also, placental dysfunction is more typically observed in early onset preeclampsia, leading to a greater need for clearance of apoptotic cells through C1q, as well as being associated with more C1q deposition (61).

Additional studies using the CBA/J X DBA/2 mouse model, described as both abortion-prone and a model for preeclampsia, have examined the role of MBL and lectin pathway in pregnancy complications (62, 63). Maternal hypertension does not occur in this model, so its usefulness as a model of preeclampsia is overstated. Affected pregnancies were characterized by high rates of fetal resorptions, intrauterine growth restriction, proteinuria and angiogenic imbalance (64). These mice do show exaggerated blood pressure response to angiotensin infusion and exercise during pregnancy compared to controls. Importantly, pregnancy failure is associated with lectin pathway activation and the terminal complement complex because MBL deficiency and anti C5 treatment in this model prevented pregnancy loss (63).

### 5.2 C3

Locally generated C3 activation products in the reproductive tract promote embryonic survival and growth. In the oviduct, C3 convertase is formed from maternal epithelial or blood-derived C3, Factor B, and Factor D, likely via the alternative pathway, resulting in formation of C3b, which is then cleaved to iC3b by local embryonic regulator Crry (rodents) or MCP (human). This locally produced iC3b acts as a potent embryotrophic factor, increasing size of the blastocyst (65–68) and supporting reproductive success. Embryonic lethality is apparent in the absence of Crry and is also consistent with impaired early pregnancy in C3 deficient mice (69). Fetal and placental weights are lower in C3 deficient mice with increased fetal resorptions and aberrant placental development including smaller spongiotrophoblast area and labyrinth.

In the BPH mouse model of superimposed preeclampsia, placental insufficiency, fetal demise and growth restriction occurs early in the pregnancy with high blood pressure in the dam appearing in later pregnancy. Two novel reagents were used to assess events early in pregnancy and inhibited the C3 convertases at the site of inflammation via targeted binding by Complement Receptor 2 (CR2): CR2-Crry and CR2-FH (51). These reagents rescued the placental morphology, fetal demise and growth restriction in the BPH mouse in early pregnancy indicating the importance of complement through C3, but the effect on maternal high blood pressure in later pregnancy was not assessed. BPH mice are hyperphagic and become obese with increased visceral reproductive white adipose tissue. Sones hypothesized that the increased reproductive white adipose tissue in the BPH mouse before and during pregnancy is a source of complement factors that contribute to the angiogenic imbalance and adverse pregnancy outcomes. They found that levels of C3 and Factor B are increased in reproductive fat in BPH mice compared to C57Bl/6 controls, suggesting the reproductive white adipose tissue may be a source of complement components influencing reproductive outcomes (70). Caloric restriction in the BPH mouse attenuated C3 expression at the maternal fetal interface early in pregnancy and restored the angiogenic imbalance to that of a control mouse. These data suggest the reproductive white adipose tissue may be a source of complement components working in a non-canonical fashion to influence reproductive outcomes but further studies are needed to determine if complement from adipose tissue is mechanistically connected to maternal hypertension and fetal development later in pregnancy (70).

### 5.3 C3a and C5a

Besides iC3b, the cleavage fragment C3a also plays an important role in embryonic development. The C3a-C3aR axis has been shown to regulate cell attraction during collective migration of neurocrest cells in zebrafish and Xenopus embryos (71, 72). Beyond development, C3a and C5a have also been evaluated as biomarkers of preeclampsia. In 1989, Haeger et al. first reported an increase in both C3a and C5a in women with preeclampsia and HELLP syndrome (73). Later, Lynch et al. showed that plasma C3a levels were elevated in early pregnancy among women who developed hypertensive disorders of pregnancy (848 vs. 757 ng/L, P<0.001) (74). Among women with obesity (BMI >30 kg/m^2^) and elevated plasma C3a levels before 20 weeks gestation, the odds of preeclampsia were significantly increased (aOR 8.8, 95%CI 3-24, P<0.001) (75). However, He et al. evaluated plasma samples as early as 6–8 weeks of gestation but found no difference in C3a or C5a levels between women who developed preeclampsia and those who did not (76). Further studies over the past decade have been mixed, in part due to variable study design and assay systems. Four studies have reported elevated plasma C3a levels in preeclampsia compared to healthy pregnancies (35, 73, 77, 78) while two found no difference (79, 80). Similarly, five studies showed increased plasma C5a levels in preeclampsia or HELLP compared to healthy controls (73, 77–80), whereas two did not (81, 82). Burwick et al. demonstrated a fivefold increase in urinary C5a levels in women with severe preeclampsia compared to matched healthy controls (80). A follow up study, evaluating the same cohort of patients, showed that urinary C5a levels did not correlate significantly with urinary albumin levels (r=0.37, P=0.08), but did correlate strongly with urinary levels of KIM-1, a marker of proximal tubule injury (r=0.60, P=0.001) (83). Investigators hypothesized that complement activation in severe preeclampsia may propagate tubular injury in the kidney, as seen in mouse models of renal ischemia, where upregulation of KIM-1 in the proximal tubule is mediated by products of complement activation (84, 85). Notably, among the subset of studies analyzing severe preeclampsia specifically, all showed significantly elevated plasma C5a levels compared to healthy controls, suggesting that terminal complement activation is more consistently associated with severe disease. Standardization of methodologies and assay systems will be critical in future complement biomarker studies in preeclampsia to improve comparability and reliability of these results.

In addition to plasma biomarkers, evaluation of amniotic fluid offers insights into local complement activity. Banadakoppa et al. measured C3a, C4a and C5a in amniotic fluid in mid-gestation and found significantly higher C3a levels in women who later developed early-onset preeclampsia (<34 weeks) compared to controls (318.7 ng/mL vs. 254.5 ng/mL, *P*=0.04), while C4a and C5a levels did not differ significantly (86).

Animal studies further support the role of C3a and/or C5a in the pathophysiology of preeclampsia. Regal et al. used a rat model of placental ischemia-induced hypertension and demonstrated that inhibition of complement activation with soluble CR1, prevented maternal hypertension but did not reverse fetal resorptions or growth restriction (50). Specific inhibition of C3aR or C5aR attenuated the hypertension induced by placental ischemia, implicating C3a and C5a in disease progression (87). Using mesenteric artery myography, the cholinergic vasodilation was impaired following the RUPP surgery, and this was rescued by the C5a antagonist but not the C3a antagonist, indicating that the complement system through C5 cleavage is important for the vascular dysfunction. Around the same time, Burwick and Feinberg (88) were the first to show that C5 blockade with eculizumab could also be used effectively to treat preeclampsia and HELLP syndrome, consistent with the protective effects seen with soluble CR1 and C5a antagonism in animal models.

### 5.4 Role of antibody in complement activation and pathophysiology of preeclampsia

Angiotensin II is a potent vasoconstrictor and binds to two major receptors: Type 1 (AT1R) and Type 2 (AT2R). Wallakut et al. initially demonstrated increased autoantibodies to the AT1R (AT1-AA) in preeclampsia compared to normal pregnancies (89). These autoantibodies are not unique to preeclampsia but are also increased in a variety of conditions such as refractory hypertension (90), aortic dissection (91), or kidney transplant rejection. AT1-AA activate, rather than block, the AT1R, and enhance angiotensin II signaling. This activation is blocked by AT1R antagonists such as losartan. Since, antigen antibody interactions are known to activate the complement system, it seems reasonable to hypothesize that complement activation in preeclampsia may be due to AT1-AA interaction with the AT1R. In humans, the AT1-AA belong to the IgG3 isotype which are strong activators of the complement system.

Several animal models have been used to investigate the role of complement and AT1-AA in preeclampsia. In one such model female rats expressing the human angiotensinogen gene are mated with males expressing the human renin gene. Human renin from the fetus crosses the placenta to cleave human angiotensinogen that cannot be cleaved by rat renin, resulting in increased angiotensin II, AT1-AA production and hypertension (92). These rats demonstrate albuminuria, IgG deposition and complement component staining (C1q, C3, C3c and C5b-9) in the kidneys, implicating complement activation in the pathophysiology. However, no direct test of complement involvement in placental pathology or resulting hypertension was performed, presenting as a clear gap in knowledge in determining a critical role for complement in this model. In a mouse model, injection of human AT1-AA induced preeclampsia-like symptoms and complement activation (93, 94). These effects were attenuated by losartan and by high doses of C3aR antagonist, SB290157 (95) suggesting the importance of C3a in the preeclampsia-like symptoms induced by injection of the human AT1-AA. However, these studies are limited by the high doses of SB290157 used that may have off-target effect and partial agonist activity. The limitations of the C3a antagonist SB290157 include C3a receptor agonist activity and cross-reactivity with C5aR2 at higher doses (96, 97). Although development of C3a antagonists remains limited and improved pharmacological tools to block the C3a receptor are still lacking (1), future studies could employ C3aR knockout mice to further investigate whether C3a plays a critical role in mediating AT1-AA–induced preeclampsia-like symptoms.

LaMarca’s group employed a similar model in the rat showing that AT1-AA injection during pregnancy results in hypertension, increased sFLT1 and oxidative stress, i.e. all hallmarks of preeclampsia. Both an endothelin A antagonist and an AT1R blockade reduced the blood pressure (98) confirming that AT1-AA interact with AT1R to cause the high blood pressure in pregnancy. AT1-AA were also detected in the rat reduced uterine perfusion pressure (RUPP) model of placental ischemia, where placental ischemia is induced on gestation day 14, resulting in hypertension and detectable AT1-AA by day 19. The hypertensive response following placental ischemia was prevented by an AT1-AA inhibitory peptide (99), implicating AT1-AA as critical. Regal used the same RUPP rat model to indicate that complement activation was critical for placental ischemia induced hypertension and verified that losartan attenuated RUPP-induced hypertension (100). However, losartan did not affect complement activation. Thus, in the rat model of placental ischemia induced hypertension, both AT1-AA and complement activation are critical mechanistic pathways. However, the evidence does not indicate that AT1-AA initiate complement activation to cause hypertension following placental ischemia.

Besides AT1-AA, placental ischemia in the rat may invoke recruitment and deposition of IgM antibodies, another potent trigger of complement activation (100). Ischemia-reperfusion studies across various organ systems have consistently implicated IgM as a key initiator of complement-mediated injury (101–104) with the specific complement pathways activated depending on the affected tissue and context. In preeclampsia, increased IgM deposition has been observed in both the placenta and kidneys of affected pregnancies (105, 106), as well as in the placental ischemia induced model of hypertensive pregnancy in rats (100). Regal et al. used the RUPP rat model and demonstrated deposition of both IgM and C3 in placenta of RUPP rats (87). Thus, placental IgM deposition following placental ischemia could be responsible for the complement activation and resultant hypertension.

The role of autoantibodies against C1q and FH in preeclampsia have also been investigated (107, 108). In a study by Dijkstra et al. (107), maternal serum samples from women with preeclampsia, healthy pregnancies, and nonpregnant women were analyzed across cohorts from the Netherlands, Finland, and Norway. No differences were found in anti-C1q or anti-FH autoantibody levels between preeclamptic and normal pregnancies. However, factor H levels were lower, particularly in early-onset cases, suggesting a role for impaired complement regulation in its development, despite no difference in circulating autoantibodies. Agostinis et al. measured C1q bound-circulating immune-complexes (CIC-C1q) in the serum of pregnant women with preeclampsia compared with healthy controls and detected higher CIC-C1q levels in preeclampsia (P=0.0004). Women with early-onset preeclampsia showed even greater CIC-C1q levels compared to those with late-onset preeclampsia. In contrast to the CIC data, preeclampsia patients had lower levels of C1q and anti-C1q compared with healthy controls (108). Study authors noted that the significant difference in anti-C1q levels among preeclampsia patients in their study, in contrast to the study by Dijkstra et al. (107), could be explained by the finding that differences in anti-C1q levels are most pronounced in the first trimester of pregnancy (108). Low anti-C1q levels in early pregnancy could reflect abnormal placentation and subsequent apoptosis occurring in the first trimester among women who later develop preeclampsia.

To further explore the importance of antibody as an important initiator of complement activation in placental ischemia induced hypertension, B cell depletion experiments were conducted using the rat model of placental ischemia induced hypertension with disparate results. While LaMarca reported that depleting rat B cells with anti-human CD20 (rituximab) reduced RUPP-induced hypertension (109, 110), Regal’s group found no such effect using anti-rat CD20 (111–113) where B cells in the blood and peritoneum were certainly depleted, but circulating IgM antibody was still apparent. Given the short window between placental ischemia and hypertension onset (GD14 to GD19), it is plausible that natural IgM, present independently of prior immunization is more relevant as an initiator of complement activation than the generation of an IgG3 autoantibody within 5 days of the placental ischemic event. In addition, rituximab targets human CD20, but not rat CD20 (113, 114), raising concerns about the specificity of B cell depletion with rituximab in rat models. Further studies are needed to determine whether specific B1 or B2 cell subsets and the antibodies they produce play a critical role in placental ischemia induced hypertension and complement activation. Such insights could better inform the development of targeted therapeutic strategies in humans.

Another strategy to explore the importance of B cells in initiating complement activation through production of antibody is the use of adoptive transfer techniques. Herrock adoptively transferred B2 cells from RUPP rats into normotensive pregnant rats and induced hypertension but did not cause an increase in circulating C3a (109, 115). In a similar model, placental CD19+ B cells from preeclamptic patients adoptively transferred into normal pregnant rats also induced hypertension along with complement activation, but a critical role for the complement activation in the increased blood pressure was not evaluated. In the RUPP rat model, Herrock et al. (116) demonstrated that an antibody to B cell activating factor (BAFF) prevented the placental ischemia induced hypertension and circulating AT1-AA, but did not significantly decrease circulating BAFF or complement activation. A surgical sham control group or isotype control injection for the anti-BAFF was not evident in this study and is a clear limitation that complicates interpretation of the data. Their data suggests that the complement activation is not initiated by the AT1-AA in this model. Since inhibition of complement activation with soluble CR1 attenuated placental ischemia induced hypertension in the rat as well (50) certainly multiple redundant pathways can drive the hypertensive phenotype.

### 5.5 C5b-9, the membrane attack complex

Five studies have demonstrated that plasma C5b-9 levels are increased in preeclampsia compared to healthy controls (35, 78, 80, 82, 117) while two studies found no difference (77, 81). Plasma C5b-9 levels are also elevated in other hypertensive disorders of pregnancy, including gestational hypertension, chronic hypertension, and preeclampsia without severe features (80, 117) suggesting that terminal complement activation (C5a and C5b-9) may be a shared feature across these conditions.

Burwick et al. first showed that urine C5b-9 levels are increased more than 4-fold among women with severe preeclampsia. This finding has been validated across three different clinical studies (80, 82, 117). In contrast to plasma C5b-9 levels, increased urinary C5b-9 levels appear to be more specific to women with severe preeclampsia (80, 117). In one study, urinary excretion of C5b-9 was detected in 96% of cases with severe preeclampsia compared to just 12% of controls with chronic hypertension and 8% of healthy controls (80). In a multicenter observational study, Burwick et al. showed that urine C5b-9 concentrations differentiated preeclampsia with severe features from other hypertensive disorders of pregnancy, with area under the receiver operating characteristic curve (ROC) of 0.74 (95% CI, 0.68–0.80) (117). Notably, 40% of women with severe preeclampsia (42/104) had urine C5b-9 concentration 22 ng/mL or greater compared with 0% (0/137) of those with chronic or gestational hypertension (P<0.001) and 11% (6/57) of those with preeclampsia without severe features (P<0.001). After multivariable logistic regression, including adjustment for total urine protein, women with urinary C5b-9 ≥22 ng/mL had tenfold increased odds of having severe preeclampsia (adjusted OR 10.0, 95% CI 3.5–29, P<0.001).

Under physiological conditions, urinary excretion of C5b-9 is not expected due to its large size (greater than 1,000,000 Daltons) (118). However, C5b-9 may form at the glomerular membrane with shedding into the urine, or C5b-9 may result from complement-mediated inflammation and cellular injury at the proximal tubule (83–85, 119, 120). Burwick et al. showed that urinary excretion of C5b-9 is increased in association with other urinary proteins such as albumin and KIM-1, indicative of glomerular and proximal tubule injury (83). Guseh et al. demonstrated that urinary excretion of C5b-9 is also strongly associated with the anti-angiogenic state in preeclampsia, characterized by plasma elevation of sFLT1 and suppression of PlGF and VEGF levels (121). Pregnant women with detectable levels of C5b-9 in the urine, compared to those without detectable levels, had significantly higher plasma levels of sFLT1 (32,029 versus 4556 pg/mL, P < 0.0001) and significantly lower levels of PlGF (15.6 vs. 226 pg/mL, P < 0.0001) and VEGF (119 vs. 153 pg/mL, P = 0.001).

In addition to systemic effects, C5b-9 deposition contributes directly to placental injury in preeclampsia. Rampersad et al. showed that under hypoxic conditions, placental trophoblast deposition of C5b-9 increases with enhanced apoptotic cell death (122). The surface density of C5b-9 staining in preeclamptic placentas is increased 2-fold compared to placentas from uncomplicated pregnancies. Subsequent studies by Banadakoppa et al. (47) and Collier et al. (48) similarly found increased trophoblast deposition of C5b-9 in placentas from women with preeclampsia and HELLP syndrome, compared to placentas from healthy pregnancy. In the study by Collier et al., the degree of placental C5b-9 staining was similar in preeclampsia and HELLP syndrome (48).

### 5.6 Adipsin (factor D)

Factor D, also known as adipsin, is a key protein in the alternative pathway, where it facilitates the cleavage of Factor B and formation of the C3 convertase (C3bBb). In addition to its immunologic role, Factor D is produced in large quantities by adipose tissue and contributes to metabolic regulation. Mice deficient in adipsin (Factor D) are fertile with no obvious abnormalities (123). However, when subjected to diet induced obesity, glucose homeostasis is impaired (124). These data suggest that without Factor D, the alternative pathway C3 convertase is not generated, resulting in reduced C3a and subsequent disruption of glucose regulation. In humans, serum levels of adipsin are significantly higher in late pregnancy in preeclampsia (125) and potentially even in early pregnancy (126) compared to normal pregnancy, suggesting it may play an important role in fat metabolism and glucose homeostasis that are altered during gestation and preeclampsia.

Preeclampsia is a condition unique to humans, making it inherently challenging to model in animals. Maternal hypertension is generally considered an essential feature for an animal model, while other characteristics, such as abnormal placental development or dysfunction, proteinuria, angiogenic imbalance, endothelial dysfunction, and fetal growth restriction are desirable to more accurately replicate the human condition. The strengths and limitations of different animal models used in pre-clinical studies have been recently reviewed (127) with no single animal model emerging as superior. In this review, we have focused on animal models of preeclampsia in which the role of complement has been investigated; however, complement involvement remains largely uninvestigated in the majority of available animal models. Collectively, these animal studies of complement in preeclampsia and pregnancy point to the complexity and heterogeneity of preeclampsia pathogenesis. This is paralleled by the human studies where evidence for complement activation differs with severity of the preeclampsia and the clinical scenarios. Consequently, a one-size-fits-all treatment approach is unlikely to be effective, highlighting the need for individualized therapeutic strategies tailored to the underlying mechanisms in each patient.

## 6 Complement regulators in pathophysiology of preeclampsia

### 6.1 Complement receptor 1

Human Complement Receptor 1 (CR1; CD35) reacts with C3b and C3b degradation products and is present on human erythrocytes, but not rodent erythrocytes. Immune complexes tagged with C3b degradation products are cleared via the reticuloendothelial system by a CR1 dependent mechanism. A study by Feinberg et al. (128) reported reduced CR1 expression in erythrocytes of preeclamptic patients compared to normal pregnancy, suggesting that the reduced clearance of immune complexes may add to the general inflammation associated with preeclampsia. This reduction in CR1 could be inherited or acquired based on studies in autoimmunity, suggesting that evaluation of genetic variations in CR1 should be investigated in preeclampsia.

### 6.2 Complement receptor 1-related gene/protein y

Mice with a homozygous deficiency in Crry exhibit embryonic lethality before 10 dpc (days post coitus) (129) whereas the heterozygote embryos survive. The death of homozygous embryos is associated with excessive C3 deposition and neutrophil infiltration. This lethality is rescued in a C3-deficient animal providing evidence that fetal demise is dependent on C3. Further studies have shown that the maternal alternative complement pathway is primarily responsible for this outcome since neither IgM nor C4 deficiency rescues the Crry homozygous knockouts, whereas Factor B deficiency does (130). In addition, C6 deficiency is ineffective in rescue indicating that the Membrane Attack Complex is not critical (131). While much has been learned from these global knockout models, recent work by Banadakoppa has refined our understanding using a placenta-specific approach. By employing intrauterine injection of shRNA (short hairpin RNA that acts as silencing RNA) to dose dependently reduce Crry in the mouse placenta at 10.5 dpc, after the critical window for complement-mediated embryonic lethality in homozygous knockouts, the researchers observed that ~ 30% reduction in placental Crry led to increased C3 deposition in the labyrinth at 17.5 dpc, decreased fetal weight and increased blood pressure in the dam (132) – symptoms indicative of preeclampsia. This data establishes the importance of placental Crry in maintaining normal fetal growth and blood pressure during pregnancy and reinforces the concept that the extent of complement activation modulates the severity of preeclampsia.

### 6.3 CD55, decay accelerating factor and CD46, membrane cofactor protein

In mice, CD55 is not expressed in trophoblasts or early embryos, and mice deficient in the GPI anchored form of DAF are fertile (133). Although DAF is detectable in the mouse placenta by 10.5 dpc (130) this timing does not align with early embryonic loss observed in Crry-knockout mice, suggesting that DAF does not play a critical role in preventing complement-mediated fetal demise. CD46, another complement regulatory protein, is expressed in mouse testes (130) and plays a role in the interaction between oocyte and sperm during fertilization (134, 135). CD46 can also act as a C3b receptor on the surface of the lymphocyte for intracellularly generated C3b (134, 135).

### 6.4 CD59, Inhibitor of membrane attack complex

Mice deficient in CD59 are healthy and fertile (136), suggesting that CD59 is not essential for a normal pregnancy. However, in preeclampsia, placental deposition of C5b-9 is increased which may be due to increased complement activation or due to impaired regulation by CD59. Buurma et al. found that placental mRNA expression of complement regulatory proteins CD55 and CD59 were increased 2-fold and 4-fold, respectively, in placentas of women delivered for preeclampsia compared to placentas from uncomplicated deliveries (105). The investigators hypothesized that upregulation of CD55 and CD59 in the face of increased complement activation suggests the presence of a feedback mechanism to maintain trophoblast integrity. An observational case control study of preeclampsia with severe features also found that plasma levels of CD59 were elevated while urinary levels were reduced, relative to normotensive controls (137). Although soluble (plasma) CD59 is less effective than its membrane-bound form, it may still contribute to systemic complement regulation in preeclampsia.

### 6.5 CSMD family

The family of CSMD (CUB and Sushi Multiple Domains) proteins includes CSMD1, 2 and 3 with high expression in the brain. The primary protein sequence of CSMD proteins is homologous with complement control proteins and they have been shown to have the ability to regulate complement activation at the C3 convertase and/or C5b-9 steps in complement activation (5, 6). With mutations in the CSMD1 protein, male and female infertility can occur (138). In addition, in CSMD1 knockout mice neurodevelopmental abnormalities occur, and altered expression of CSMD1 occurs in the peripheral blood of schizophrenic patients (139). CSMD3 knockout progeny have neurodevelopmental abnormalities consistent with autism, as well as fetal growth restriction similar to offspring of preeclamptic pregnancies.

## 7 Genetics of complement dysregulation in preeclampsia

As described, disruptions in the complement system can compromise a normal pregnancy and increase the risk of preeclampsia. In some individuals, genetic mutations in complement proteins contribute to this dysregulation. The process is analogous to that seen in atypical hemolytic uremic syndrome (aHUS), a complement-mediated disease, characterized by endothelial injury and thrombotic microangiopathy (TMA), leading to kidney failure, stroke and cardiovascular complications (140). aHUS occurs due to loss-of-function mutations in complement regulators (FH, FI, MCP) or gain-of-function mutations in complement proteins (C3, Factor B) (141). Similarly, in preeclampsia, genetic variants in complement regulators and proteins have been identified suggesting a shared pathophysiological pathway involving complement-mediated vascular injury (142–145).

Gene-based burden testing for rare and unique variants (minor allele frequency <1%) in the FINNPEC cohort identified five heterozygous rare variants in *CFH* (L3V, R127H, R166Q, C1077S and N1176K) in 7 women with severe preeclampsia (32). Functional characterization revealed that four of these variants resulted in a quantitative (low factor H levels) or a qualitative (normal FH level but low function) defect, resulting in dysregulation of the complement system. More recently, rare missense variants in C5 and C6 were also discovered in the FINNPEC cohort (146). Two variants in C5 and one in C6 were associated with increased risk of developing preeclampsia (OR = 24.13 and OR= 22.75). In yet another study that genotyped 622 women with preeclampsia and 2027 controls (without preeclampsia) from the FINNPEC and National FINRISK cohorts, variants in complement receptor 3 (CR3) and complement receptor 4 (CR4) were identified (147). These variants demonstrated altered binding to iC3b suggesting that changes in complement-facilitated phagocytosis may contribute to pathogenesis of preeclampsia. A study from nephrology centers in Belgian and France examined 11 patients with preeclampsia and reported that four (36%) caried mutations in genes regulating the alternative pathway of complement activation (142). Another prospective study (PROMISSE, Predictors of pRegnancy Outcome: bioMarkers In antiphospholipid antibody Syndrome and Systemic Lupus Erythematosus) identified heterozygous mutations in MCP, CFI, or CFH in 7 of 40 patients (18%) with preeclampsia and coexisting systemic lupus erythematosus and/or antiphospholipid antibody syndrome (143). Five of these patients had risk variants in MCP or CFI that were previously identified in aHUS. One had a novel mutation in MCP that was functionally characterized and demonstrated impaired regulation of C4b. The association of variants of MCP and CFI was also confirmed in a cohort of non-autoimmune preeclampsia patients in which five of 59 were heterozygous for mutations. A recent retrospective study further highlights the clinical impact of complement variants. Among 68 kidney transplant recipients diagnosed with a TMA between 2008 to 2023, 93% underwent genetic testing for complement variants (148). Compared to 3467 transplant recipients without TMA, 42 of the 68 patients were found to carry genetic variants or acquired autoantibodies and were diagnosed with a complement-mediated TMA. Strikingly, half of these individuals had a history of hypertension or preeclampsia as the underlying cause of end stage kidney disease. Of these, 78.5% experienced recurrent TMA and graft loss shortly after kidney transplantation. Together, findings from human genetic studies and experimental mouse models suggest that defective regulation of the complement system allows for the excessive complement activation that leads to placental damage, abnormal placental development, generalized endothelial activation, and the release of antiangiogenic factors toxic to the fenestrated endothelium of glomeruli, the choroid plexus, and liver sinusoids. Ultimately, these events contribute to the breakdown of maternal-fetal tolerance and development of clinical preeclampsia.

## 8 What have we learned from manipulating complement during pregnancy?

Complement therapeutics have been used successfully to treat diseases characterized by excessive complement activation, including antiphospholipid syndrome (APLS), paroxysmal nocturnal hemoglobinuria (PNH), atypical hemolytic uremic syndrome (aHUS) and lupus, and this has included pregnant individuals. The only C5 inhibitor with safety data for use in pregnant and lactating women is eculizumab, a humanized monoclonal antibody against C5, which inhibits cleavage of C5 into C5a and C5b and prevents formation of the terminal complement complex C5b-9 (149). It was approved by the Food and Drug Administration to treat paroxysmal nocturnal hemoglobinuria (PNH) in 2007 and aHUS in 2011, due to its therapeutic efficacy in both conditions (150, 151).

Eculizumab has provided benefit for women with PNH during pregnancy, as evidenced by a high rate of fetal survival and a low rate of maternal complications (152). PNH erythrocytes which lack the complement regulatory proteins CD55 and CD59 are more susceptible to complement-mediated intravascular hemolysis. Pregnancy-associated PNH is linked to worse neonatal and maternal morbidity and fetal and maternal mortality rates of 4-9% and 8-12% respectively (153). Danilov et al., 2009 first described initiation of eculizumab in the third trimester of pregnancy for women with PNH (154). Among pregnant women with PNH treated with eculizumab, Kelly et al. found that eculizumab was detectable in 7 of 20 cord blood samples (range 11.8 to 21.1 ug/ml), suggesting that it crosses the placenta at low levels. While IgG monoclonal antibodies are transported across the placenta through the FcRn receptor, eculizumab was engineered with human IgG2 and IgG4 heavy-chain sequences to form a hybrid constant region with reduced affinity for Fc receptors (126, 129). Hallstensen et al. showed that treatment with eculizumab during pregnancy does not appear to alter the complement system activity of the newborn (103). Kelly et al. also examined 10 breast milk samples among women receiving eculizumab and the drug was not detected in any of the samples, leading the authors to conclude that breastfeeding is safe (114). However, continued vigilance is warranted given the small sample size. Use of eculizumab for PNH in pregnancy has been demonstrated in numerous other case reports showing favorable pregnancy outcomes (155). More recently there have been case reports of favorable pregnancy outcomes in women who received eculizumab from conception to delivery (156–161).

Antiphospholipid syndrome (APS) is a systemic autoimmune disorder characterized by development of antiphospholipid antibodies (aPLAb) which include lupus anticoagulant, anticardiolipin antibodies and anti-beta2 glycoprotein I (162). It is estimated that aPLAbs are associated with 50,000 pregnancy losses every year in the US (163). The clinical manifestations can be classified as thrombotic, obstetric or catastrophic. A hallmark of obstetric APS is pregnancy complications, which include preeclampsia, recurrent early pregnancy loss, fetal death or premature birth due to intrauterine growth restriction (164). Lupus anticoagulant positivity has been shown in several prospective studies to be a strong predictor of poor pregnancy outcomes. Patients who carry all 3 autoantibodies (triple aPLAb positivity) are reported to have significantly worse pregnancy outcomes with increased risk of fetal loss. Approximately 1% of patients with APS may develop catastrophic APS (CAPS) which is a severe and life-threatening form characterized by microvascular and macrovascular thrombosis that develop simultaneously or over a short period of time leading to multiorgan failure and a significantly increased risk of mortality. Based on the documented crosstalk between complement and coagulation pathways, activation of complement could be an adjunctive mechanism that could explain the pathogenic effects of aPLAb and the frequent failure of anticoagulation treatment (165). Women with APS and low complement levels are more likely to experience hypertensive disorders during pregnancy, pregnancy loss, shorter duration of pregnancy and higher frequency of late fetal growth restriction. In the PROMISSE study, Bb and C5b-9 levels at 12-15 weeks of pregnancy were predictive of adverse pregnancy outcomes in women with SLE and/or aPLAb (143). This study suggested that the presence of aPLAb leads to activation of the alternative pathway, resulting in an increased frequency of obstetric complications. Overall, the evidence supports the role of complement in aPLAb-mediated obstetric morbidity regardless of SLE. Analysis of the CAPS registry revealed outcomes from 39 patients treated with eculizumab, of whom 74.4% went into remission without relapse (166). Various case reports have demonstrated the potential of eculizumab in preventing APS-related complications in pregnant women at high risk of thrombosis or CAPS (167–169).

Pregnancy related aHUS is reported to occur in 1 in 25,000 pregnancies accounting for 7% of all TMA cases with 79% of these presenting in the postpartum period (170). Pregnancy (particularly the postpartum period) is considered one of the major triggers for aHUS or complement-mediated TMA (CM-TMA). Pregnancy is an immunologically privileged condition where placental damage is prevented by regulators of complement activation, the levels of which increase during pregnancy to assist in the clearance of placental fragments that enter the maternal circulation, as a result of syncytiotrophoblast turn-over. Reversal of these phenomena in the postpartum period is speculated to predispose to development of aHUS/CM-TMA (36). Prior to the approval of C5 inhibitors, treatment with plasma exchange was the mainstay for the management of CM-TMA although the overall efficacy was poor. The rationale behind this treatment is to reduce the quantity of a mutant protein by plasmapheresis and then deliver a functionally normal protein by plasma infusion. Plasma therapies still remain the initial treatment of choice in countries where complement inhibitors are not available, or the cost precludes their use (171). However, the use of anti-complement therapies, such as eculizumab, has been associated with improved outcomes including decreased progression to end stage kidney disease, less time on dialysis, and successful remission of disease (172).

Fakhouri et al. analyzed data from the Global aHUS Registry to evaluate renal survival according to time to ESRD, for patients with and without eculizumab treatment in women with pregnancy-triggered aHUS (p-aHUS, n=51) compared to women of childbearing age with aHUS and no identified trigger (non-p-aHUS, n = 397) (173). Eculizumab led to significantly improved renal outcomes in women with aHUS; adjusted hazard ratio for time to ESRD was 0.06 (p = 0.006) in the p-aHUS group and 0.20 (p < 0.0001) in the non-p-aHUS group.

In 2019, Lu et al. described a pregnant woman who was diagnosed with aHUS at 22 weeks gestation and initiated on eculizumab treatment. At 24 weeks gestation she was also diagnosed with superimposed preeclampsia and eculizumab treatment was continued, with prolongation of pregnancy by an additional 25 days. In retrospective laboratory analyses, the authors found that the sFLT1-PlGF ratio decreased after initiation of eculizumab and they hypothesized that eculizumab may have moderated the antiangiogenic activity in preeclampsia (174). In 2020, Lokki et al. described a patient who developed postpartum aHUS after delivery for preeclampsia and HELLP syndrome at 34 weeks gestation (175). The patient developed severe renal failure requiring dialysis, but she quickly recovered after the diagnosis of aHUS was made and treatment with eculizumab was initiated.

A newer longer acting C5 inhibitor, ravulizumab is also approved for CM-TMA. Ravulizumab has been engineered from eculizumab by changing four amino acids (176). This change preserves the binding of ravulizumab to C5 in serum but allows it to dissociate from C5 in the acidified endosome (pH 6.0). Additionally, these amino acid alterations also result in an increased efficiency of neonatal Fc receptor-mediated recycling of ravulizumab, thereby, leading to an increased half-life of ~52 days compared to ~11 days for eculizumab (**Table 2**). Due to the structural modifications noted above, ravulizumab has much greater affinity for the neonatal Fc receptor compared to eculizumab, potentially enabling higher uptake into the fetal circulation and breastmilk, but safety data is lacking. The use of ravulizumab in post-partum aHUS/CM-TMA has been reported in a subgroup analysis of 8 patients from a phase 3 multicenter trial of treatment-naïve adult patients with aHUS (177). All patients presented with acute severe medical emergency associated with pregnancy or delivery (two with preeclampsia, one with gestational diabetes, two with placental abruption and five were on dialysis). Complete TMA response (defined as LDH normalization, platelet count normalization and ≥25% improvement in serum creatinine from baseline) was observed in 7 out of 8 patients in 31 days and all patients on dialysis came off dialysis within 21 days. A recent abstract presentation reported on a series of 5 PNH patients and 6 pregnancies treated with ravulizumab (178). Authors noted that ravulizumab was given for treatment of PNH throughout pregnancy, with one preterm delivery at 35 weeks and 5 term deliveries at 38–42 weeks, with no fetal abnormalities noted. Four of the patients continued lactation while on ravulizumab, with no neonatal abnormalities reported. However, no data on cord blood or breast milk levels of ravulizumab were reported, and more studies are needed to establish safety and efficacy.

**Table 2:** Comparison of anti-C5 antibodies.

Characteristic	Eculizumab	Ravulizumab
Structure	Humanized IgG2/4k monoclonal antibody against C5 (149)	Humanized IgG2/4 hybrid monoclonal antibody against C5 with two histidine substitutions at complementarity-determining region, and two amnio acid substitutions to increase affinity for human FcRn (176)
Half-life, mean (SD)	11.3 (3.4) days (200)	49.7 (8.9) days (201)
Fc Engineering for FcRn Binding	No FcRn engineering beyond natural IgG properties	Engineered FcRn region to enhance binding at acidic pH (~6.0) and release at neutral pH (~7.4) (176)
Lysosomal degradation	The eculizumab-C5 complex is not expected to dissociate efficiently in the endosome and likely undergoes both lysosomal degradation and recycling (176)	Engineered to enhance the dissociation rate of the ravulizumab:C5 complex in the acidic early endosome relative to the slightly basic pH of blood, thus enhancing lysosomal degradation of the previously bound C5 and recycling of the unbound antibody (176)
Human cord blood data	Detected at low levels (88, 152)	No data
Human breast milk data	Has not been detected in breast milk samples (88, 152)	No data

No reports on the use of ravulizumab for the treatment of preeclampsia or HELLP syndrome are available. Several other drugs for CM-TMA are also in development or undergoing clinical trials. These novel complement inhibitors include crovalimab (anti-C5), nomacopan (anti-C5), pegcetacoplan (anti-C3) and iptacopan (anti-factor B), and narsoplimab [mannose-binding lectin-associated serine protease 2 (MASP-2) (179).

## 9 Complement inhibition in preeclampsia and HELLP

Burwick and Feinberg were the first to show that C5 blockade with eculizumab could be used effectively to treat preeclampsia and HELLP syndrome (88). They described a previously healthy pregnant woman who developed severe preeclampsia and HELLP syndrome at 26 weeks of gestation. Following off-label use of eculizumab, there was resolution of hemolysis and thrombocytopenia, and normalization of liver enzymes. Maternal and fetal status improved sufficiently to prolong pregnancy an additional 17 days, allowing more time for fetal maturation and decreasing the likelihood of neonatal morbidity. In a subsequent report, the authors reported that the concentration of C5b-9 in blood and urine decreased in conjunction with C5 blockade and disease remission (180). While there was a good clinical outcome, the authors noted persistent elevation of C5a following C5 blockade, and they cautioned that extrinsic factors such as thrombin may also generate C5a independent of the C5 convertase (181). Finally, they also cautioned that eculizumab or eculizumab-C5 complexes may cross the placenta, or deposit in the maternal kidney, and long-term effects are unknown (182–184).

More recently in 2025, Gerber et al. reported on 3 cases of preeclampsia with severe features treated with eculizumab. All three patients had severe hypertension, proteinuria, severe fetal growth restriction, and liver enzyme elevation at time of enrollment. In addition to standard of care, study participants received eculizumab infusion at 25 6/7 weeks, 26 3/7 weeks, and 27 6/7 weeks gestation, with delivery after 32hrs, 59hrs, and 25hrs, respectively (185). No adverse maternal outcomes and no fetal or neonatal deaths were seen, but all three neonates did experience complications related to prematurity. There was not a clear maternal or fetal benefit in these 3 cases. Other case reports have also been published proposing eculizumab as a treatment for postpartum preeclampsia and HELLP syndrome complicated by progressive kidney failure (156), and non-preeclampsia TMA presenting in early pregnancy (157, 158). Prospective, randomized controlled trials are definitely needed to investigate the utility of these therapies in preeclampsia or HELLP syndrome.

## 10 Proposed complement evaluation in preeclampsia and HELLP syndrome

Treatment of preeclampsia with severe features or HELLP syndrome using a complement inhibitor such as eculizumab remains investigational and off-label. However, it is important to investigate the role of complement in severe cases of preeclampsia, especially when the maternal condition does not improve following delivery, as this may suggest thrombotic thrombocytopenic purpura (TTP) or aHUS (186). Additionally, women who have experienced severe preeclampsia or HELLP syndrome often desire guidance regarding recurrence risk for future pregnancy. If diagnostic evaluation reveals evidence of complement dysregulation, or an underlying complement gene mutation, these patients need appropriate genetic counseling and a well-defined plan prior to subsequent pregnancy.

**Figure 3:**
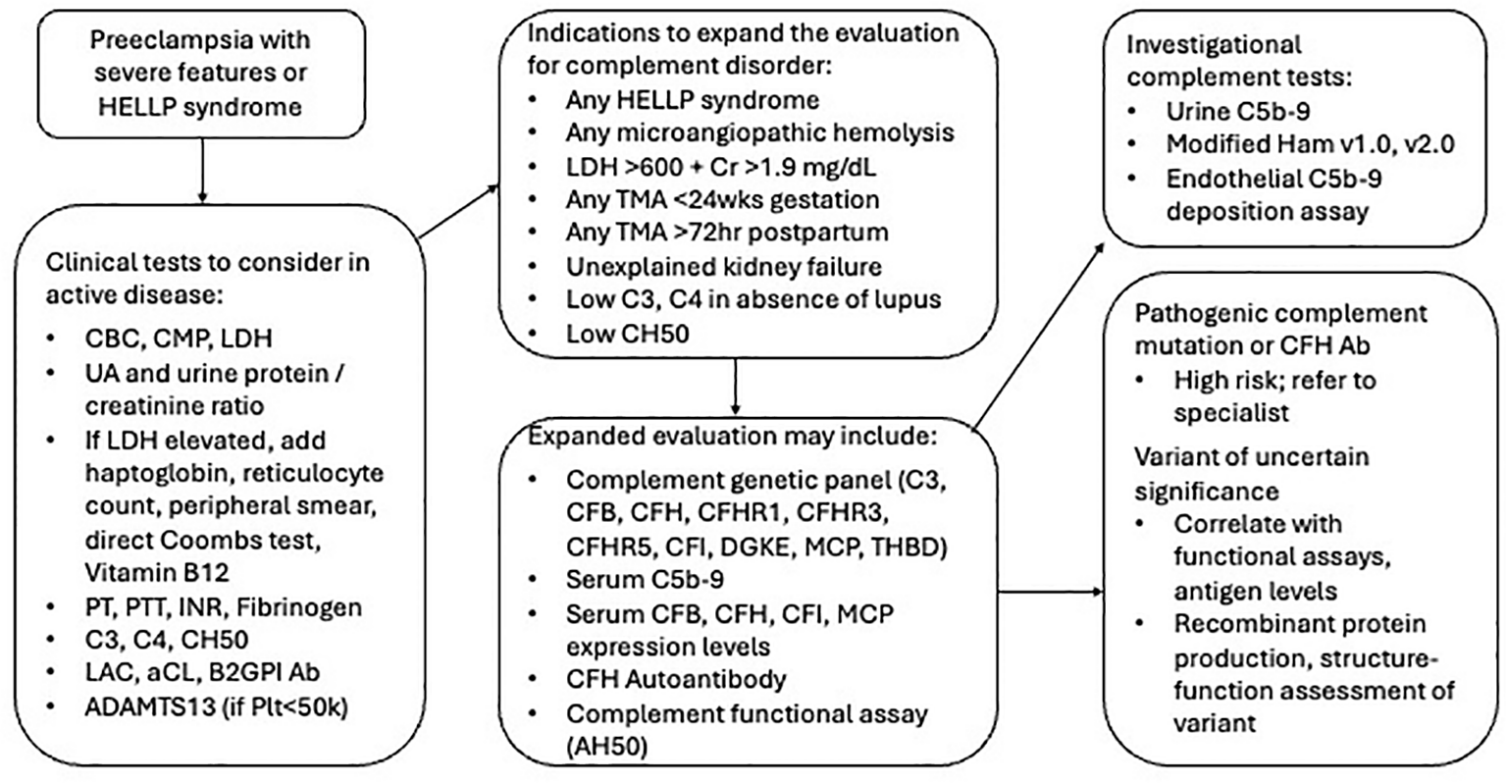
Flow diagram for proposed evaluation of preeclampsia with severe features and HELLP syndrome. Ab, Antibody; AH50, alternative complement pathway activity; CBC, complete blood count; CFB, complement factor B; CFH, complement factor H; CFI, complement factor I; CH50, total complement activity; CMP, complete metabolic panel; Cr, creatinine; HELLP, hemolysis elevated liver enzymes low platelet count; INR, international normalized ratio; LDH, lactate dehydrogenase; Plt, platelet count; PT, prothrombin; PTT, partial thromboplastin time; TMA, thrombotic microangiopathy; UA, urinalysis.

When patients present acutely with preeclampsia, especially when the disease is early-onset or severe in nature, it is critical for clinicians to determine if TMA is present (**Figure 3**). In the setting of preeclampsia, clinicians usually assess hemoglobin, platelet count, liver function (ALT, AST), and serum creatinine, but these tests are insufficient to rule out hemolysis. To determine if microangiopathic hemolysis is present, clinicians should order additional tests such as serum lactate dehydrogenase (LDH) level, haptoglobin, direct Coombs test, reticulocyte count and peripheral smear. In the setting of red cell hemolysis, LDH is usually elevated while the haptoglobin level is usually low or undetectable. In microangiopathic hemolysis, the direct Coombs test is negative, and the peripheral smear may show schistocytes. It is important to note that schistocytes may not be immediately present in acute disease, and their absence does not rule out active TMA (187). Generally, TMA is suspected in the setting of microangiopathic hemolysis, thrombocytopenia, and end-organ injury. In preeclampsia and HELLP, end-organ injury from TMA may include systemic hypertension, proteinuria, acute kidney injury, hepatic dysfunction, pulmonary edema, severe headache, or seizure, among others (188, 189).

When women present with severe preeclampsia or HELLP syndrome, it is often important to rule out other TMA disorders which may present similarly (186, 190). If there is TMA in conjunction with severe thrombocytopenia and platelet count less than 50,000/µl, the diagnosis of TTP should be ruled out through assessment of the ADAMTS13 level. The diagnosis of acquired TTP is confirmed by an ADAMTS13 activity level <10% and the presence of ADAMTS13 IgG, while congenital TTP (cTTP) is diagnosed when ADAMTS13 activity level is <10% in the absence of ADAMTS13 IgG and confirmed by mutational analyses (191). The normal physiologic alteration of pregnancy may unmask cTTP by further depleting already low ADAMTS13 levels. Thus, patients with cTTP may not manifest any clinically significant symptoms until the physiologic stress of pregnancy is underway. If there is TMA in conjunction with elevated serum creatinine, low GFR, and/or severe oliguria, the diagnosis of aHUS should be considered (33). In a study of 91 postpartum patients with either HELLP syndrome or aHUS, the diagnosis of aHUS was more likely in those with serum creatinine ≥1.9 mg/dL and LDH ≥600 U/L (specificity 100%, sensitivity 97%, *J* statistic 0.97), as this combination was seen in 91% of aHUS cases compared with 0% of HELLP cases (*P*<0.001) (192).

Even in the absence of aHUS, the evidence presented earlier in this manuscript demonstrate that preeclampsia and HELLP may be complement mediated in many cases. Thus, there is a rationale to perform a complement evaluation in severe cases, to determine if complement activation or dysregulation played a role in the disease. Clinicians may begin with readily available tests such as C3, C4 and CH50, however these tests are limited in nature. C3 and C4 are usually decreased in the setting of active lupus, due to immune complex formation, but they may be normal in preeclampsia, HELLP, or aHUS despite ongoing complement activation (since C3 and C4 are also acute phase proteins). CH50, a measure of total complement activity, is broadly elevated in acute inflammatory conditions and is often elevated even in normal pregnancy. Thus, it is less useful in determining if a disease was complement-mediated or not. Research studies noted above have shown that serum and urinary C5b-9 levels can be elevated in the setting of preeclampsia, with urinary levels being most useful (80, 117). However, urine C5b-9 testing is not routinely available for clinical use, limiting its immediate application. Serum C5b-9 can be ordered, usually as a send-out test, and may be the most accessible test for confirming that terminal complement activation was present at the time of active disease. There is an unmet need for urgent development and validation of serum and/or urine-based assays that can help with rapid diagnosis and identification of patients with complement-mediated disease so timely treatment can be initiated.

Various cell-based assays have also been used to evaluate the degree of complement activation in patients with known or suspected aHUS, and these have also been evaluated in women with preeclampsia and HELLP syndrome (193–196). While such assays are not yet validated for clinical use in pregnancy, research studies have shown promise. Vaught et al. showed that complement-mediated cell lysis is increased when the serum of women with either aHUS, severe preeclampsia or HELLP syndrome is mixed with paroxysmal nocturnal hemoglobinuria (PNH) -like reagent cells lacking GPI-anchored complement regulatory proteins (GPI-AP) (193). This assay, termed the modified Ham test (mHam), evaluates the degree of complement-mediated cell killing by measuring cell viability when patient serum is exposed to GPI-AP cells. Venou TM, et al. found that modified Ham was elevated in 36% of patients with early-onset severe preeclampsia compared to 0% of patients with healthy pregnancy (196). In another study, most patients with a diagnosis of HELLP had a positive mHam test (62%) compared to the healthy control group (16%), with a statistically significant difference between the two groups (p < 0.001). The results were similar in the group of patients with aHUS versus healthy controls (88% versus 16%, p < 0.001) (145). Recently, Cole M, et al. described an array of cell-based complement “biosensors” by selective removal of complement regulatory proteins (CD55 and CD59, CD46, or a combination thereof) in an autonomously bioluminescent HEK293 cell line. These biosensors have been presented as a sensitive method for diagnosing CM-TMA and monitoring therapeutic complement blockade but needs to be thoroughly validated clinically before being accepted for widespread use (197). This cell-based complement biosensor approach has been incorporated into the newest modified Ham 2.0 test, which is commercially available, but is limited because it has not yet been tested or validated for general use or for use in pregnancy.

Endothelial-cell derived assays for complement activation have also been assessed, in which patient’s serum is collected and mixed with resting or ADP-activated human microvascular endothelial cells (HMECs). In such assays, serum taken from patients with increased complement activation (e.g., aHUS) will demonstrate increased C5b-9 deposition on resting HMECs compared with control serum. Using this technique, Palomo et al. demonstrated that C5b-9 deposition on endothelial cells is increased following exposure to plasma from women with either aHUS, severe preeclampsia or HELLP syndrome (195). Youssef et al. also showed greater endothelial C5b-9 deposition in preeclampsia (194). Notably, the magnitude of complement-mediated hemolysis in the mHam test, and the degree of endothelial C5b-9 deposition in the HMEC assay, respectively, were similar in patients with preeclampsia, HELLP or aHUS, suggesting that aberrant complement activation is a common feature of these disorders (193, 195). Many of the above assays are conducted by and available in specialized labs. However, significantly more data and studies are needed to confirm the validity and reproducibility of these assays for clinical use in pregnancy.

Evaluation for underlying genetic variants in complement proteins should also be conducted in women with severe forms of preeclampsia or HELLP syndrome, or in those with recurrent preeclampsia in multiple pregnancies. Due to the turnaround time in such testing, and subsequent evaluation required, genetic testing will most often be utilized in counseling for long-term health, including the possibility of future pregnancies. The clinically validated complement disease next-generation sequencing-based panel consists of 15 genes (*ADAMTS13, C3, CD46, CFB, CFH, CFHR1, CFHR2, CFHR3, CFHR4, CFHR5, CFI, DGKE, THBD, MMACHC*, and *PLG*). If a well-described pathogenic variant is detected, this would confirm a role for complement in the patient’s clinical disease and may also suggest that the patient has a higher risk for recurrent preeclampsia or HELLP syndrome, though data is limited. Other variants of uncertain significance may be identified. Interpretation of such variants may require additional functional assays, biomarker testing, or, in select cases, recombinant protein production followed by structure–function assessment of the variants. Functional hemolytic assays, such as CH50 and AH50, as well as testing for antigenic levels of complement proteins (e.g., CFH, CFI) should be used to evaluate the presence of a quantitative defect. Combined genetic, antigenic, functional, and structural evaluations will help to define the role and extent of complement involvement in preeclampsia and will further facilitate the stratification of patients for targeted therapy in the future. Since this testing can take 4–6 weeks, it should not delay immediate treatment or delivery decisions for preeclampsia and HELLP syndrome in most cases, outside of investigational clinical trials. However, complement evaluation can help define the underlying pathophysiology and facilitate long term treatment decisions.

## 11 Future pregnancy

It is well known that preeclampsia has a high risk for recurrence in subsequent pregnancies, estimated overall at 20% (198), but recurrence is likely higher in those with early-onset or severe disease. Thus, women experiencing severe preeclampsia or HELLP syndrome should be offered a work-up for an underlying complement disorder as described above. While the risk for recurrent preeclampsia in the setting of an underlying complement gene mutation is unknown, data has shown that women with aHUS have high risk for recurrence in subsequent pregnancy up to 60-70% (199). Pregnancy is a well-known trigger for complement-mediated disorders, and women with prior preeclampsia and an underlying complement gene variant may warrant heightened surveillance in pregnancy. Prior to pregnancy, such patients should be referred to specialists in high-risk pregnancy, preferably those with a background in complement-mediated disorders. The care plan for future pregnancy, or decisions regarding surrogacy, will be patient-specific and should consider clinical history and other comorbidities. The monitoring plan in future pregnancy should include close surveillance for recurrent TMA and complement activation, with potential for targeted complement inhibition if complement mediated TMA is detected.

## 12 Discussion

Preeclampsia is clearly a multifaceted syndrome which poses significant risk to both mother and fetus. Its heterogeneous nature, driven by placental dysfunction, underscores the need for improved diagnostics and therapies. The complement system, a cornerstone of innate and adaptive immunity, is intricately involved in pregnancy, balancing placental development and immune tolerance and contributing to preeclampsia when dysregulated.

In healthy pregnancies, tightly regulated complement activation at the maternal-fetal interface, facilitated by regulators like CD46, CD55, CD59 and Crry (in rodents), supports trophoblast invasion and spiral artery remodeling, ensuring fetal tolerance. Dysregulation, however, triggers excessive inflammation, placental ischemia, and anti-angiogenic imbalances, central to preeclampsia’s pathophysiology, including severe forms like HELLP syndrome. Preclinical studies, such as C1q-deficient mouse models, reveal impaired placentation and preeclampsia-like features, while models like the BPH mouse demonstrate complement-driven inflammation and fetal loss, mitigated by inhibitors like CR2-Crry. Human studies associate elevated plasma and urinary C3a, C5a, and C5b-9 with severe preeclampsia. Genetic analyses identify rare variants in complement regulators (CFH, CFI, MCP) and proteins (C5, C6), suggesting a shared mechanism between preeclampsia and complement-mediated disorders like atypical hemolytic uremic syndrome (aHUS).

Therapeutically, complement inhibitors like eculizumab, a C5 blocker, show promise in case reports for severe preeclampsia and HELLP, prolonging pregnancy by reducing C5b-9 deposition. Its established safety in pregnancy for PNH and aHUS supports further exploration, while newer agents like ravulizumab and other novel complement inhibitors, require investigation. The complexity of preeclampsia’s subtypes necessitates personalized approaches, as complement’s role varies across clinical scenarios. Opportunities for future work are significant. Diagnostic testing could advance through validating cell-based assays, which detect complement-mediated injury in preeclampsia, potentially enabling early risk stratification. Developing accessible, reproducible complement biomarker and antigenic assays could enhance clinical utility. Clinical trials, particularly randomized controlled studies, are critical to evaluate the efficacy and safety of complement inhibitors across preeclampsia subtypes, addressing variable responses seen in case studies. Investigating non-canonical complement roles and species-specific differences will refine translational research.

In summary, the complement system is a pivotal player in preeclampsia, with dysregulation driving placental and systemic pathology. Advances in biomarkers, genetics, and therapeutics offer hope, but rigorous diagnostic validation and clinical trials are essential to translate these findings into effective, tailored interventions for this complex syndrome.
